# Endoscopic Management of Recurrent Anastomotic Biliary Stricture Following Deceased Orthotopic Liver Transplantation

**DOI:** 10.3390/jcm14072198

**Published:** 2025-03-24

**Authors:** Esteban Fuentes-Valenzuela, Marina De Benito Sanz, Irene Peñas-Herrero, Félix García-Pajares, Carmen Alonso-Martín, Carolina Almohalla Álvarez, Antonio Martínez-Ortega, Ramon Sanchez-Ocana, Carlos de la Serna-Higuera, Gloria Sánchez-Antolín, Manuel Perez-Miranda

**Affiliations:** Gastroenterology Department, Hospital Universitario Rio Hortega, 47012 Valladolid, Spain; efuenval@gmail.com (E.F.-V.);

**Keywords:** liver transplantation, endoscopic treatment, anastomotic stricture

## Abstract

**Background/Objectives:** Data on the natural history and endoscopic treatment outcomes of recurrent anastomotic biliary stricture (RABS) after orthotopic liver transplantation (OLT) are limited. This study aimed to evaluate the incidence and outcomes of RABS after OLT. **Methods:** A retrospective single-center study on OLT patients who underwent successful endoscopic treatment of ABS was conducted. The incidence of RABS, risk factors for recurrence, and outcomes of repeat endoscopic therapy were recorded. **Results:** A total of 131 OLT patients with ABS underwent endoscopic treatment, of which 119 successfully completed an endoscopic treatment course. After a median follow-up of 51.5 months (IQR 18.5–86.25) from ABS resolution, 26/119 patients (22.7%) developed RABS. All patients with RABS underwent a second endoscopic treatment course; 24 patients received self-expandable metal stents and 2 received plastic stents. Re-treatment was successful in 21 patients (80.8%) after a median of 8.5 months (IQR 5.25–14.50) and a total of 62 ERCPs. Adverse events occurred in two patients (7.4%)—one bacteremia and one suprastenotic biliary stricture. After a median follow-up of 65.5 months (IQR 20.75–125.5) from stent removal, only one patient had a second recurrence, which was treated with a Roux-en-Y hepaticojejunostomy. Multivariate analysis showed that older age at ABS diagnosis (OR 1.1; 95% CI: 1.1–1.2 *p* = 0.04) was the only independent risk factor for recurrence. **Conclusions:** RABS affects more than 20% of patients after successful endoscopic treatment. A second endoscopic therapy with covered self-expandable metal stents is a safe and effective option and should be considered before more invasive options.

## 1. Introduction

Endoscopy has become the initial approach to anastomotic biliary strictures (ABSs) after orthotopic liver transplantation (OLT). Endoscopic retrograde cholangiopancreatography (ERCP) with the placement of either multiple plastic stents or a single covered self-expandable metal stent (cSEMS) is the standard endoscopic treatment approach. Multiple plastic stent and cSEMS placements have comparable clinical success rates. However, cSEMSs have gained favor due to their ability to reduce the number of required procedures and lower associated costs [1,2]. Recently, cSEMSs have been proposed by clinical guidelines as the preferred type of stent for the treatment of ABS after OLT [3].

Nevertheless, cSEMSs carry a higher risk of migration. Efforts to decrease the risk of cSEMS migration include dedicated antimigration cSEMSs [4,5,6], dedicated short intraductal cSEMSs [7], or the placement of coaxial double pig-tail anchoring plastic stents [8]. Despite improvements in stent technology, the risk of ABS recurrence remains a clinical challenge, with rates ranging from 18 to 47.5% of OLT recipients [2,9]. A wider range of variation has been reported for the incidence of RABS in post-OLT patients who undergo percutaneous management, with recurrence rates ranging between 4.2 and 44% [10,11,12].

The evolution and treatment options for recurrent anastomotic biliary stricture (RABS) have not been extensively studied. Two small retrospective studies with limited follow-up have reported the incidence of RABS in OLT recipients and the treatment outcomes following multiple plastic stent endotherapy [1,13]. Alazmi et al. reported an 18% RABS rate after a median of 5 months post-OLT [1]; Dai et al. found a 25.5% RABS rate after a median of 4 months [13].

Although there are no prospective studies available on RABS management, most guidelines recommend using SEMS for initial endoscopic therapy of post-OLT ABS [3,14]. Despite this current recommendation, there is a lack of studies evaluating RABS treatment outcomes in OLT patients who primarily underwent SEMS-based endotherapy for their ABS.

Therefore, our study aimed to evaluate the incidence of RABS, risk factors for recurrence, and treatment outcomes [5].

## 2. Materials and Methods

### 2.1. Design

A retrospective case series study was conducted. Consecutive deceased brain donor OLT recipients with successfully treated duct-to-duct ABS who underwent ERCP between November 2001 and June 2022 were retrieved from a prospectively maintained endoscopy database at Hospital Universitario Rio Hortega. OLT recipients who successfully completed an endotherapy course, whether with multiple plastic stents, cSEMSs, or combination of both plastic and cSEMSs, were eligible for inclusion. Their records were reviewed for baseline features, procedural details, and treatment outcomes.

Patients with non-anastomotic strictures or bilioenteric anastomosis, those lost to follow-up, or those who did not complete the endoscopic treatment due to death or re-transplantation were excluded.

The local institutional review board approved the study protocol (22-PI046) and all subjects gave written informed consent before endoscopic procedures.

### 2.2. Participants

Consecutive OLT patients who developed ABS and were treated endoscopically with plastic or SEMS with complete follow-up data available were reviewed. Patients were followed from OLT until RABS and thereafter until death or October 2023 at the Liver Unit clinic.

### 2.3. Procedures

All endoscopic procedures were performed at a high-volume advanced and referral endoscopy unit (>1200 ERCPs/year) using standard duodenoscopes by experienced endoscopists. Antibiotic prophylaxis with 4/0.5 g of piperacillin/tazobactam (or ciprofloxacin/metronidazole in case of allergy) was routinely administered. The procedure was performed under endoscopist-directed sedation using propofol. Biliary cannulation was followed by cholangiography and biliary sphincterotomy in patients with either a native papilla or a stenosed prior sphincterotomy. Balloon dilation was performed occasionally at the endoscopist’s discretion before cSEMS or plastic stent placement using 8 or 10 mm biliary balloon dilating catheters (Hurricane^®^, Boston Scientific, Natick, MA, USA). Finally, a single cSEMS (Wallflex®, Wallstent® models, from Boston Scientific or BCS Hanarostent®, BCG Hanarostent® models, from M.I. Tech, Seoul, Republic of Korea) or multiple plastic biliary stents (CLSO plastic biliary stent from Cook Endoscopy, Limerick, Ireland) were placed with the proximal end 2 cm above the ABS and the distal end across the papilla. Moreover, 8 to 10 mm diameter cSEMSs were preferentially placed provided that the bile duct was >6 mm in diameter. The maximal number of plastic stents placed was at the endoscopist’s discretion and eventually related to the bile duct diameter.

### 2.4. Study Outcomes and Definitions

The primary outcome was the rate of RABS among OLT recipients who completed an initial endoscopic treatment course for ABS. An endoscopic treatment course involved periodic ERCP for stent exchange/upsizing at scheduled intervals until definitive ABS calibration. RABS was defined as the reappearance of an isolated fibrotic stricture localized within one centimeter of the surgical anastomosis on the endoscopic retrograde cholangiogram in patients with objective clinical, laboratory, and imaging signs of biliary obstruction.

Secondary outcomes included risk factors for RABS, endoscopic procedure details, RABS endotherapy success rates, duration of repeat endoscopic treatment courses, and secondary recurrence rates following successful RABS endotherapy.

### 2.5. Patient Follow-Up

Patients were regularly followed at the Liver Clinic every 3–6 months with abdominal ultrasound and routine blood tests. If suspicion arose about possible ABS or RABS, magnetic resonance imaging was undertaken, which was followed by ERCP for confirmation and treatment if warranted. During endoscopic treatment courses for ABS and RABS, stent exchanges were planned at 4–8 months intervals for cSEMSs and 3–4 months intervals for plastic stents. The dominant interval for planned cSEMS removal changed during the study period. During an early period of cSEMS-based endotherapy of ABS at HURH between 2005 and 2013, the first revision of ERCP was planned at 4–6 months after stent placement [15], whereas, between 2014 and 2022, the first revision of ERCP was planned at 6–8 months [6]. Patients who underwent endoscopic treatment for RABS were followed up until death or October 2023.

### 2.6. Statistical Analysis

Continuous variables are presented as the mean and standard deviation and as the median with interquartile range (IQR), as warranted. Categorical variables are presented as numbers and percentages. Chi-square was used as a comparative test for categorical variables in this study. Student’s *t* test and Mann–Whitney U test were used for the comparison of continuous variables with normal distribution and non-normal distribution, respectively.

Kaplan–Meier analysis with log-rank test was used to evaluate survival among patients developing RABS and time until RABS. Uni- and multivariant analyses were performed to identify RABS risk factors.

The statistical analyses were performed using Stata (StataCorp. Stata Statistical Software: Release 16. College Station, TX, USA: StataCorp LP). A *p* < 0.05 was considered statistically significant.

## 3. Results

### 3.1. Patients Characteristics

A total of 131 OLT patients with ABS underwent endoscopic therapy. Of these, 119 patients (81.5% male) successfully completed their initial course of endotherapy. Eleven patients died before completing the endotherapy course, while the remaining patient required retransplantation due to a relapse of the primary liver disease. Detailed baseline characteristics are provided in Table 1.

### 3.2. Recurrence of Anastomotic Biliary Stricture

**Figure 1:**
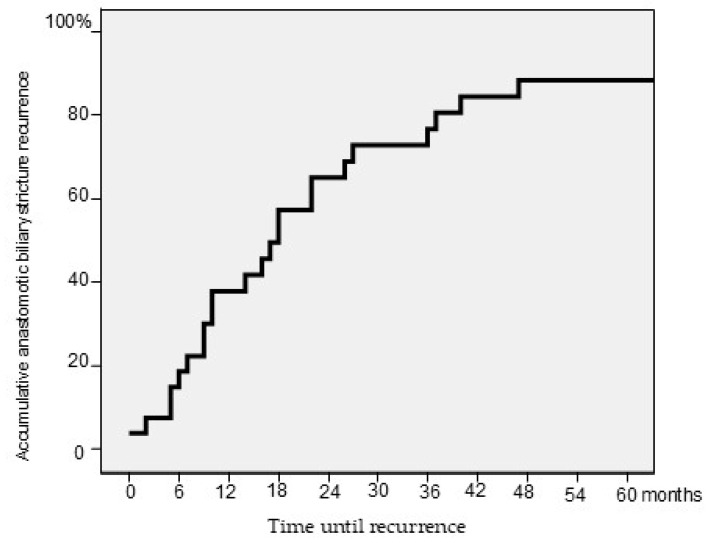
Kaplan–Meier analysis including the median time until anastomotic biliary stricture recurrence among all patients developing recurrent anastomotic biliary stricture.

**Figure 2:**
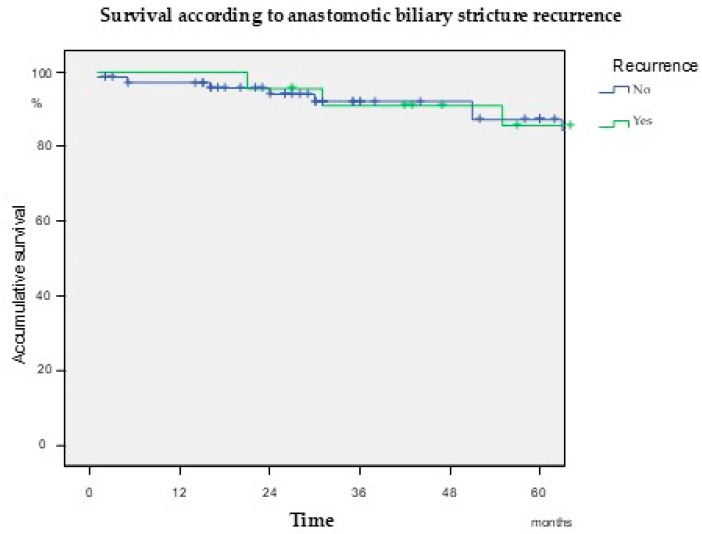
Kaplan–Meier analysis evaluating overall survival according to the development of recurrent anastomotic biliary stricture. Log-rank *p* = 0.76.

After a median follow-up of 51.5 months (IQR 18.5–86.25), 26 patients (22.7%) developed RABS. The median time from initial ABS resolution to RABS diagnosis was 17 months (IQR 7–36). Thirty-eight percent of patients presented with RABS during the first year after ABS resolution (Figure 1). Importantly, overall survival was comparable between patients with and without RABS (Figure 2). The median age in the RABS group was 64 years (IQR 55–69.5), with decompensated cirrhosis being the primary indication for OLT in 21 patients (80.8%), followed by hepatocellular carcinoma in 5 (19.2%).

The clinical presentation of RABS varied: 57.7% of patients developed acute cholangitis, 38.5% had isolated transaminase elevation, and one patient presented with bacteremia. Only one patient (3.8%) was in the intensive care unit due to biliary sepsis at the time of the index ERCP for RABS treatment (Table 2). Additionally, a total of four patients developed non-anastomotic strictures (3.4%).

### 3.3. Treatment of RABS

ERCP was attempted in all 26 patients with RABS and was technically successful in 25 (96.2%). One patient required endoscopic ultrasound (EUS)-guided biliary drainage after failed guidewire passage into the donor bile duct. EUS-guided choledochoduodenostomy was performed by placing a cSEMS into the donor bile duct; elective magnetic compression anastomosis of the disconnected bile duct was then successfully performed.

**Figure 3:**
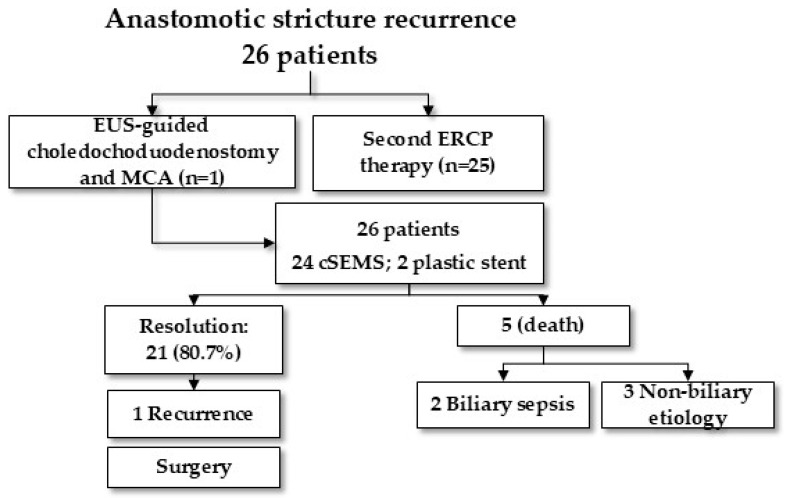
Treatment evolution of anastomotic biliary stricture recurrence. EUS: endoscopic ultrasound; MCA: magnetic compression anastomosis; ERCP: endoscopic retrograde cholangio-pancreatography; SEMS: self-expandable metal stent.

The distal magnet was placed transpapillarily into the recipient bile duct, and the proximal magnet was placed through the mature choledochoduodenostomy tract into the donor bile duct, as detailed elsewhere [16]. After magnet coupling, both magnets were removed 8 days later, and a cSEMS was placed across the reconnected duct with the patient returning to the standard ERCP treatment protocol (Figure 3).

Plastic stents were placed in just 2 (7.7%) patients; 24 patients (92.3%) were treated with cSEMS. A median of 2 ERCPs (IQR 2–3) with a median of 1 stent placement (IQR 1–2) were required. Sphincterotomy was performed in 12 patients (44.4%) during the index ERCP. Additional balloon dilation was required in 10 patients (38.5%) with a median of 1 dilation session (IQR 1–1.25).

**Figure 4:**
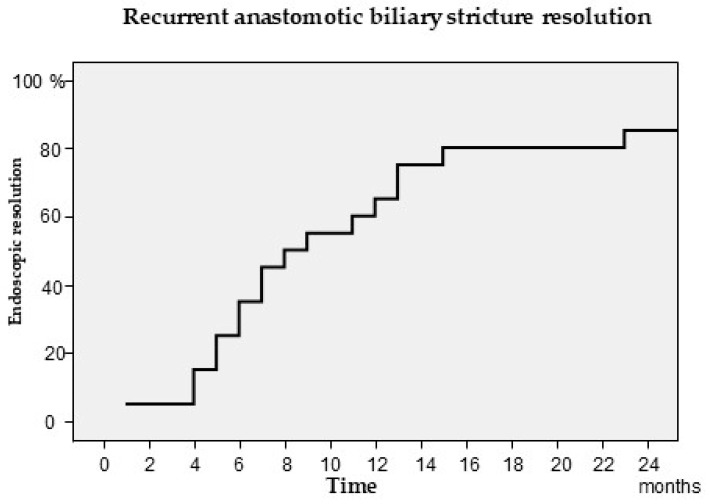
Kaplan–Meier assessing the time until endoscopic resolution of the recurrent anastomotic biliary stricture.

RABS resolution was observed in 21 patients (80.8% treatment success rate on an intention-to-treat basis; 100% per-protocol success rate) after a median of 8.5 months (IQR, 5.25–14.50) (Figure 4). A total of 62 ERCPs (median 2 ERCPs, IQR 2–3) were performed and a total of 36 stents were placed. Eleven (52.4%) patients had RABS resolution at the first endoscopic revision, nine (34.6%) patients had RABS resolution at the second endoscopic revision, and the remaining patients had RABS resolution at the third endoscopic revision. Stent removal was successful in all patients. Five patients died during the course of ongoing endotherapy, accounting for all treatment failures (100% treatment success rate on a per-protocol basis). Two deaths were due to biliary-related sepsis, one patient had bilateral pneumonia, one patient had post-OLT non-Hogdkin lymphoma, and one patient had cerebral hemorrhage.

Post-procedure adverse events were identified in two patients (7.4%), one patient with bacteriemia, and another patient with stent-induced suprastenotic biliary stricture. Stent obstruction was identified in four patients (14.8%). Overall, stent migration was observed in eight patients (29.5%), with three patients (11.1%) showing complete migration during the first endoscopic revision.

After a median follow-up of 65.5 months (20.75–125.5), only one patient presented a second RABS, which was eventually managed surgically with a Roux-en-Y hepaticojejunostomy.

### 3.4. Risk Factors

Univariate analysis revealed that previous prolonged endotherapy (>6 months) (OR: 2.78; 95% CI 1.1–7.2; *p* = 0.037) and age at ABS diagnosis (OR 1.1; 1.0–1.1; *p* = 0.038) were risk factors for RABS. However, via multivariate analysis, only older age at ABS diagnosis (OR 1.1; 95% CI: 1.1–1.2 *p* = 0.04) was identified as an independent risk factor for RABS (Table 3).

## 4. Discussion

This retrospective analysis of OLT recipients with ABS treated with cSEMS or plastic stents demonstrated a recurrence rate of 22.7% after an extended follow-up period, distinguishing this study from prior reports. This lengthy follow-up is particularly valuable for evaluating the long-term development of biliary strictures. Despite this extended observation, a second course of endotherapy proved highly effective in over 80% of cases, with a low incidence of adverse events.

The reported RABS rates in the literature vary widely, ranging from 0 to 37% [17]. Alazmi et al. reported a recurrence rate of 18% after a median follow-up of 10 months, while another study identified a 25.5% recurrence after a median of 4 months [1,13]. Both studies included patients treated with plastic stents for primary treatment. In contrast, our study with a notably longer follow-up and a significant proportion of patients treated with SEMS yielded a similar RABS rate. Notably, the recurrence in our cohort occurred later, with a median time of 17 months, underscoring the importance of long-term follow-up in detecting late recurrences, though 40% of patients experienced RABS within the first year. This early recurrence raises concerns regarding the initial resolution of ABS.

To date, only one study has examined the efficacy of a second endotherapy course. Sun-Chan et al. achieved an efficacy rate of 83.3% in a cohort of 24 patients treated with plastic stents for RABS after a median of four endoscopic sessions [13]. In our study, cSEMS was predominantly used for both initial ABS and RABS treatment, potentially explaining the lower median number of ERCPs required. Our findings align with current guidelines for post-OLT biliary strictures, suggesting that cSEMSs may offer the most appropriate management strategy for RABS [3].

Beyond endoscopic therapy, other treatment options have only been assessed in patients with ABS. A meta-analysis including 33 studies assessed the efficacy of endoscopic treatment for ABS in OLT recipients. It found a variable need for surgical or percutaneous interventions [2]. A recent retrospective study of 449 patients identified a 21% incidence of biliary complications, with only 5.6% requiring non-endoscopic approaches [18].

Surgical resection of the stenotic segment, duct-to-duct reconstruction, and conversion to a hepaticojejunostomy or retransplantation have been described as alternatives [19]. Robotic or open hepaticojejunostomy has shown excellent results for late ABS, although the morbidity may be higher than an endoscopic therapy [20]. In our study, only one patient required surgery after a second episode of RABS. Consequently, an endoscopic approach should be the first choice for any recurrence, reserving surgical options for cases refractory to endotherapy after a failed second course of endotherapy.

Our study has several limitations that should be acknowledged and considered when interpreting the results. Firstly, the retrospective design inherently limits the ability to establish causality and introduces the possibility of selection and information biases, despite efforts to minimize them through the use of a prospectively maintained database.

Our study lacks a control group for surgical or percutaneous interventions; however, this limitation is partly mitigated by the robust evidence supporting the safety and efficacy of ERCP as the first-line treatment for biliary strictures in liver transplantation recipients. Current clinical guidelines and numerous studies have established ERCP, particularly with the use of cSEMS, as a highly effective and minimally invasive option for managing biliary strictures. Given this strong body of evidence, it would be ethically challenging to justify the inclusion of a surgical control group, as surgical approaches such as hepaticojejunostomy are associated with greater morbidity and should be reserved for cases refractory to endoscopic management. This context underscores the relevance of our findings, which align with the established role of ERCP in this setting and further support its application for recurrent anastomotic biliary strictures.

Although the sample size for RABS (*n* = 26) may appear small and limit the statistical power to identify additional risk factors in the multivariate analysis, it is important to highlight that published evidence on the management of recurrent anastomotic biliary strictures remains scarce. To date, few studies have specifically addressed this challenging clinical scenario, and even fewer have provided detailed long-term follow-up data. While the marginal significance of older age as a risk factor should indeed be interpreted with caution, we believe that describing these findings contributes valuable insights to the limited body of literature. By reporting these results, our study provides practical data that may guide clinical decision-making and lay the groundwork for future research on the management of RABS in liver transplant recipients.

Future studies addressing these limitations with larger, controlled, and prospective designs will be essential to confirm and expand upon our findings.

In conclusion, RABS is a prevalent issue that impacts more than twenty percent of previously treated patients. In this scenario, a second endotherapy course with cSEMSs appears to be a safe and effective option and should be considered as the preferred option before trying any other more invasive therapy. However, larger, controlled, and prospective studies are required to determine the most appropriate endoscopic therapy for RABS.

## Figures and Tables

**Table 1:** Baseline characteristics of patients who completed a first endotherapy course. IQR: interquartile range; SEMS: self-expandable metal stent; PSC: primary sclerosing cholangitis; HBV: hepatitis B virus; HCV: hepatitis C virus.

	*n* = 119 Patients
Gender (male)	97 (81.5)
Median age, years (IQR)	58 (50–63)
OLT indication	33 (27.7%)
Hepatocellular carcinoma	79 (66.4%)
Child B-C	6 (5%)
Acute liver failure	1 (0.8%)
Others	
Etiology	
Alcohol	61 (51.3%)
HCV	28 (23.5%)
PSC	11 (9.2%)
HBV	6 (5%)
Other	13 (10.9%)
Cold ischemia time, minutes (IQR)	350.5 (325–428.8)
Type of stent	
cSEMS	56 (47.1)
Plastic stent	20 (16.8)
Combined	43 (36.1)
Additional balloon dilation	36 (30.3)
Stent indwell time, months (IQR)	6 (4–8)

**Table 2:** Baseline characteristics amongst patients developing recurrent anastomotic biliary stricture. IQR: interquartile range; SEMS: covered self-expandable metal stents; AST: aspartate aminotransferase; ALT: alanine transaminase; GGT: gamma-glutamyl transferase, ALP: alkaline phosphatase.

	*n* = 26 Patients
Gender (male)	20 (76.9%)
Median age, years (IQR)	64 (55–69.5)
OLT indication	
Hepatocellular carcinoma	5 (19.2)
Child B-C	21 (80.8)
Cold ischemia time, minutes (IQR)	350 (317.5–455)
Type of stent	
cSEMS	24 (92.3%)
Plastic stent	2 (7.7%)
Additional balloon dilation	10 (38.5%)
Sphincterotomy	11 (42.3%)
Stent dwelling time, months (IQR)	8.5 (5.25–14.5)
AST, units/L, median (IQR)	70 (47–92.5)
ALT, units/L, median (IQR)	99 (39.5–168.5)
GGT, units/L, median (IQR)	370 (171.5–786.5)
ALP, units/L, median (IQR)	304 (155–446)
Total Bilirubin, mg/dL, median, (IQR)	2.1 (1.2–7.9)

**Table 3:** Uni- and multivariate analysis for risk factors for recurrent anastomotic biliary stricture.

	Univariate Analysis			Multivariate Analysis		
	OR	95% CI	*p* value	OR	95% CI	*p* value
Prolonged previous endotherapy (>6 months)	2.78	1.1–7.2;	0.037	6.7	0.8–54.3	0.07
Age at ABS diagnosis	1.1	1.0–1.1	0.038	1.1	1.1–1.2	0.04
>1 stent during previous endotherapy	0.83	0.3–2.1	0.702	0.5	0.2–1.4	0.2
ABS during first 30 days	0.62	0.1–2.9	0.55	0.9	0.2–5.6	0.9

## Data Availability

The raw data supporting the conclusions of this article will be made available by the authors upon reasonable request.

## Abbreviations

The following abbreviations are used in this manuscript:

RABS	Recurrent anastomotic biliary stricture
ABS	Anastomotic biliary stricture
ERCP	Endoscopic retrograde cholangiopancreatography
cSEMS	Covered self-expandable metal stent
OLT	Orthotopic liver transplantation
